# Electrochemical Protection of Cyanobacterial Cells from Molecular Oxygen Enables Sustained PhotoH_2_ Production

**DOI:** 10.1002/anie.202519077

**Published:** 2026-01-02

**Authors:** Panpan Wang, Florian Paul, Marko Boehm, Jens Appel, Kirstin Gutekunst, Wolfgang Schuhmann, Felipe Conzuelo

**Affiliations:** ^1^ Analytical Chemistry – Center for Electrochemical Sciences (CES) Faculty of Chemistry and Biochemistry Ruhr University Bochum Universitätsstr. 150 D‐44780 Bochum Germany; ^2^ Molecular Plant Physiology Bioenergetics in Photoautotrophs University Kassel Heinrich‐Plett‐Straße 40 D‐34132 Kassel Germany; ^3^ Instituto de Tecnologia Química e Biológica António Xavier Universidade Nova de Lisboa Av. da República Oeiras 2780‐157 Portugal

**Keywords:** Biophotovoltaics, Cyanobacteria, Hydrogen evolution, Photosynthesis, Redox polymers

## Abstract

Photosynthetic hydrogen (photoH_2_) production is appealing for sustainable energy conversion. Oxygenic photosynthesis uses water as the sole electron source and light to lift electrons to a high energy level. The energized electrons are used by the hydrogenase for the catalytic conversion of protons into H_2_. Photosynthetic microorganisms own all enzymatic equipment for this process, and the feasibility of photoH_2_ production was demonstrated. However, one of the main limitations is that O_2_, which is generated as a byproduct of photosynthesis, compromises the activity of most hydrogenases and hinders the wider applicability of this strategy. We tackle this challenge, showing the protection of cyanobacterial cells from metabolically‐generated O_2_ by the integration of intact cells into a viologen‐modified redox polymer. Electrochemical activation of the redox polymer allows O_2_ removal in proximity to the cyanobacterial cells with a steep diffusional gradient of O_2_ outside the cells. Microelectrochemical local analysis of O_2_ and H_2_ confirms the protection and the possibility of photoH_2_ production. Moreover, the use of mutant cells integrating a photosystem I‐hydrogenase fusion enables sustained photosynthetic H_2_ production under these conditions, with the electrons for prolonged photoH_2_ production most likely originating from photosynthetic water splitting.

## Graphical abstract

Photosynthetic microorganisms can be used to perform H_2_ evolution driven by light. However, a crucial limitationlies in the associated generation of molecular oxygen as a by‐product of photosynthesis, which compromises the activity of the biocatalyst performing H2 production, i.e., the hydrogenase. To tackle this challenge, we present an electrochemical O_2_‐scavenging strategy, allowing efficient O_2_ removal from immobilized microbial cells.

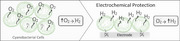

## Introduction

Photosynthetic microorganisms offer the potential to use solar energy as a sustainable approach for clean hydrogen production.^[^ ^1^, ^2^, ^3^ ^]^ These microorganisms own a highly specialized light‐harvesting machinery coupled to a complex photosynthetic electron transport chain. Electrons extracted from water photo‐oxidation at the manganese cluster of photosystem II (PSII) are later pumped to high energy levels by the subsequent action of light‐dependent reactions taking place at PSII and photosystem I (PSI). The high‐energy electrons can then be transferred to a hydrogenase (H_2_ase) for catalytic hydrogen production with high efficiency.^[^ ^4^ ^]^ Although cyanobacteria and green microalgae contain H_2_ase enzymes, typically, these photosynthetic microorganisms do not exhibit photoH_2_ production under illumination as the energized electrons derived from photosynthesis are diverted into metabolic routes as, for example, the Calvin–Benson–Bassham (CBB) cycle for CO_2_ fixation, for sustaining cell growth and function.^[^ ^5^ ^]^ Only residual H_2_ is produced from the release of excess reductants under fermentative dark conditions.^[^ ^2^, ^6^ ^]^ Photosynthetic H_2_ (photoH_2_) evolution effectively takes place only for brief periods under anoxic conditions shortly after the transition between dark and illumination, prior to the activation of CO_2_ fixation.^[^ ^3^, ^6^, ^7^, ^8^ ^]^ However, the H_2_ produced is subsequently taken up again by the cell, using it as a temporary store of surplus electrons.^[^ ^9^ ^]^ In order to enhance H_2_ production rates and to abolish uptake of photoH_2_, aiming at biotechnologically relevant photoH_2_ production, different factors limiting the efficiency of the process need to be taken into account.^[^ ^10^ ^]^ To overcome the challenges associated with the use of photosynthetically derived electrons by competing metabolic processes and photoH_2_ uptake,^[^ ^7^, ^11^, ^12^ ^]^ a direct fusion of H_2_ase to PSI in vivo was established via genetic manipulations in algae and cyanobacteria.^[^ ^13^, ^14^ ^]^ These mutants show prolonged photoH_2_ evolution, but electron transfer between PSI and H_2_ase must still be optimized.

The most severe and challenging limitation of the process is the O_2_‐sensitivity of H_2_ases.^[^ ^3^, ^13^, ^14^, ^15^ ^]^ One approach to overcome this hurdle is the isolation of PSI–H_2_ase fusions for in vitro assays that could be protected from O_2_ by the implementation of two electrochemical half cells, decoupling water oxidation and H_2_ evolution.^[^ ^16^ ^]^ In addition, there are attempts to express oxygen‐tolerant NiFe–H_2_ases in cyanobacteria; however, they are hampered by the fact that these H_2_ases do not accept electrons from ferredoxin and produce no photoH_2_.^[^ ^17^ ^]^ In vitro systems using these H_2_ases coupled to PSI require a laborious preparation protocol and may suffer from short operational lifetimes^[^ ^18^ ^]^ as the isolated protein complexes lack the natural repair and maintenance mechanisms available in living photosynthetic cells. Moreover, as the photosynthetic process uses water as the electron source, in vivo systems can offer a more attractive, economical, and long‐lasting approach for photoH_2_ production, which may allow a sustained operation facilitating the potential fabrication of large‐scale devices. The challenge of using whole cells for photoH_2_ production in vivo is that O_2_ generated during photosynthesis, from water photo‐oxidation at PSII, compromises the performance of the H_2_ase. Cyanobacterial H_2_ases are inactive in the presence of O_2_ but can be reactivated within seconds upon O_2_ removal.^[^ ^9^, ^15^, ^19^ ^]^ Several strategies have been proposed to decrease O_2_ production so as to create the conditions necessary for H_2_ase activity. In case of green algae, sulfur deprivation, CO_2_ limitation, and the addition of O_2_ scavengers have been used to prolong the photoH_2_ production phase.^[^ ^20^, ^21^, ^22^ ^]^ In contrast to this, an appreciable effect of sulfur depletion on photoH_2_ production could not be demonstrated in cyanobacteria^[^ ^23^ ^]^ and these approaches require extensive treatment of the cultures that precludes their biotechnological application. Downregulation of PSII and activity impairment, involving the use of PSII inhibitors, has been shown to diminish O_2_ production and thus decrease intracellular O_2_ concentrations.^[^ ^24^ ^]^ However, lowering water splitting rates at PSII has the negative impact of limiting electron availability to PSI and subsequently to H_2_ase, ultimately constraining solar‐to‐hydrogen production rates.

**Figure 1:**
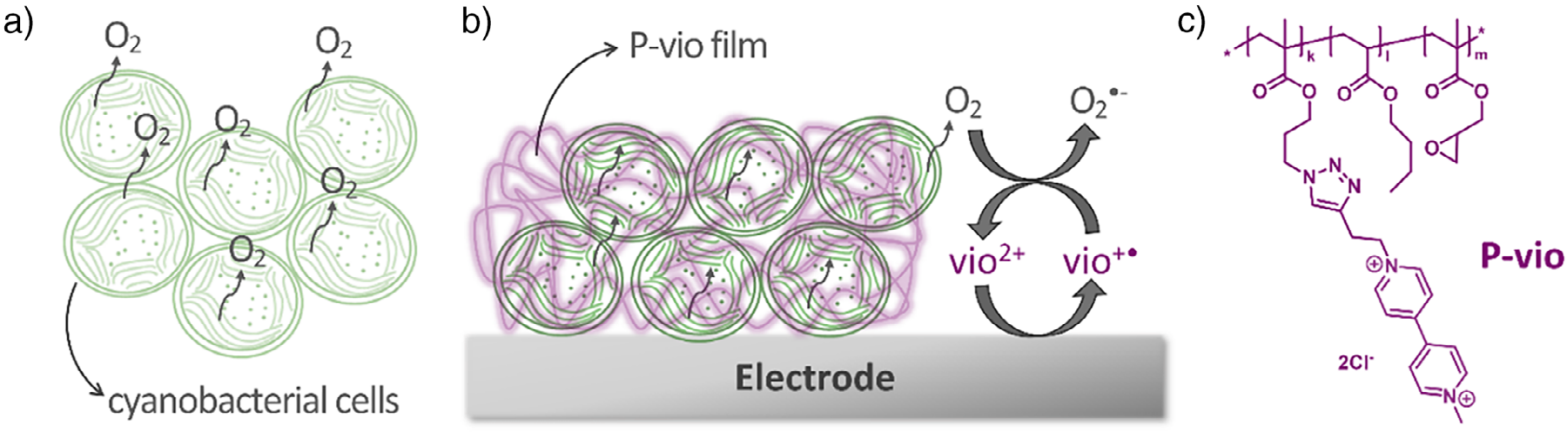
a) Cyanobacterial cells release O_2_ under illumination as photosynthesis proceeds. b) Proposed scheme for protection of cyanobacterial cells from released O_2_ by integration into a viologen‐modified polymer (P‐vio) deposited on an electrode. Reduced viologen moieties generated electrochemically efficiently scavenge molecular oxygen within the film. c) Chemical structure of the viologen‐modified redox polymer.

To overcome these limitations, we propose the implementation of an electrochemical O_2_‐removal system, which ensures the necessary conditions for H_2_ase activity to realize photoH_2_ production in vivo using cyanobacterial cells. The proposed strategy involves the integration of cyanobacterial cells in a viologen‐modified redox polymer deposited on an electrode (see Figure 1). In this way, the cells are entrapped in a hydrogel matrix for ease of manipulation of the photoH_2_ production system. Moreover, by applying an adequate potential to the electrode, the viologen moieties at the redox polymer are reduced and can efficiently consume O_2_ that is generated during photosynthesis. As the redox polymer film surrounds the cells, O_2_ is efficiently depleted within the film and a diffusional gradient is concomitantly established, forcing O_2_ out of the cells. To investigate this approach in detail, the release of O_2_ and H_2_ is locally investigated under illumination using scanning electrochemical microscopy (SECM),^[^ ^25^ ^]^ allowing for the evaluation of the correlation between light‐driven photosynthetic activity and H_2_ production.

## Results and Discussion

### Inactivation of H_2_Ase by O_2_ in Liquid Cultures

**Figure 2:**
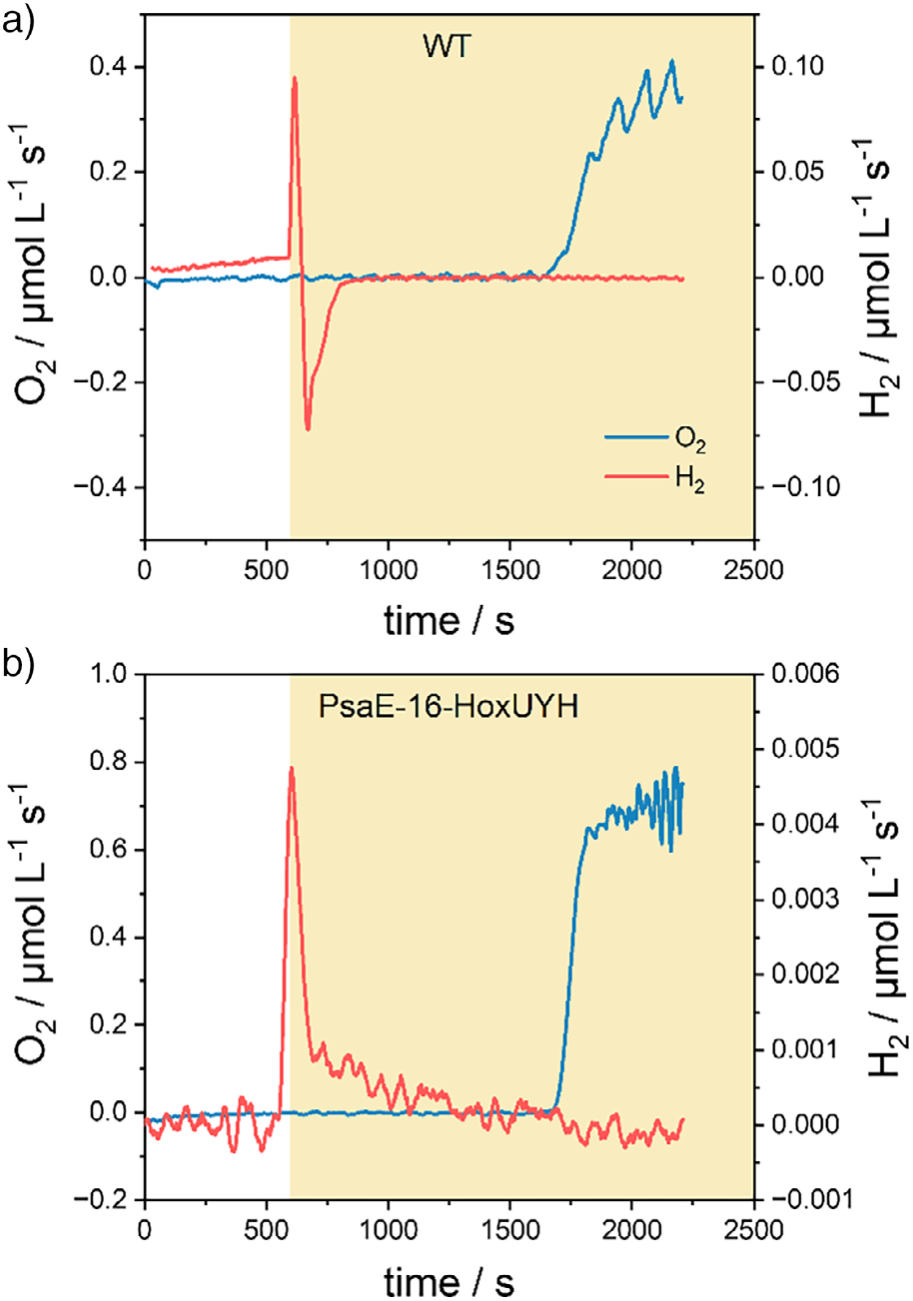
Simultaneous measurements of the rates of O_2_ and H_2_ turnover in wild‐type cells (a) and cells of the PsaE‐16‐HoxUYH mutant (b). O_2_ was measured by a membrane‐inlet mass spectrometer (MIMS), while H_2_ was measured with a specific microsensor. Rates were calculated over a period of 30 s (for details see Experimental Section in the Supporting Information).

Wild‐type (WT) cells of the cyanobacterium *Synechocystis* sp. PCC 6803 and the PSI–H_2_ase fusion mutant PsaE‐16‐HoxUYH were investigated. For a schematic representation of the fusion construct, please refer to Figure S1. The conventional approach to producing photoH_2_ over extended periods in liquid cultures involves the use of glucose together with an O_2_‐consuming enzyme mix consisting of glucose oxidase and catalase. One disadvantage of this approach is that glucose is also metabolized by the cyanobacterial cells. Electrons from glucose oxidation are thus fed into the photosynthetic electron transport chain and might ultimately be used for photoH_2_ production. The electrons for photoH_2_ production, therefore, no longer come exclusively from water splitting at PSII, as desired. WT and PsaE‐16‐HoxUYH were initially characterized in conventional preparations using glucose and the enzymatic O_2_ scavenging system. An experimental setup that allows the measurement of O_2_ by a membrane‐inlet mass spectrometer (MIMS) was used, while H_2_ production was monitored with a specific H_2_ sensor placed in the same sample. To initiate photoH_2_ production, the cells were submitted to anaerobiosis by adding glucose, glucose oxidase, and catalase in the dark. After 10 min dark incubation, the light was switched on. In WT cells (Figure 2a), photoH_2_ evolution was directly followed by H_2_ uptake by the native bidirectional H_2_ase (HoxEFUYH). In the fusion mutant PsaE‐16‐HoxUYH, photoH_2_ uptake was no longer possible due to the lack of complete diaphorase subunits (HoxEFU), as it was observed earlier for other PSI–H_2_ase mutant strains.^[^ ^14^ ^]^

In PsaE‐16‐HoxUYH a sharp decrease in the rate of H_2_ evolution was still visible after the onset of light, falling to zero as soon as O_2_ production becomes apparent (Figure 2b). The first decline in H_2_ production could most likely be ascribed to competition with CO_2_ fixation. Due to the concentrations chosen for the experiment, glucose should be gradually consumed, and the enzymatic O_2_‐scavenging reaction should become increasingly limited over time, resulting in a steadily increasing O_2_ concentration as detected by MS. This is mirrored by the slowly declining rate of H_2_ evolution in the second phase of photoH_2_ production. PhotoH_2_ production ceases completely as soon as O_2_ accumulates.

### Feasibility of the O_2_‐Scavenging Approach

The newly proposed O_2_‐scavenging strategy is based on the immobilization of cyanobacterial cells within a viologen‐modified redox polymer (P‐vio). This eliminates the need to add the enzyme‐based O_2_‐scavenging system (glucose oxidase and catalase) that requires the use of glucose, which is an undesirable electron donor. Instead, the system should depend exclusively on water splitting at PSII for providing the electrons that are ultimately used for H_2_ production. Glassy carbon electrodes were first modified with WT cells integrated into P‐vio and characterized electrochemically. Voltammetric measurements revealed the characteristic wave for the redox interconversion of the polymer‐tethered viologen moieties (Figure S2), with a midpoint potential of about −275 mV versus SHE, in agreement with previous work.^[^ ^26^, ^27^, ^28^ ^]^ Moreover, the obtained responses were found to be stable over several potentiodynamic cycles, confirming the stability of the film integrating the cyanobacterial cells.

Electrochemically‐assisted O_2_ consumption proceeds through its reduction by means of viologen radical cations (vio^•+^) generated at applied potentials more negative than the midpoint potential of the vio^++^/vio^•+^ couple, i.e., *E_app_* < −275 mV versus SHE. The reduction of O_2_ proceeds through the generation of hydrogen peroxide (H_2_O_2_), which is further reduced to H_2_O. However, as it has been identified before,^[^ ^29^ ^]^ the kinetics for O_2_ reduction are faster than those for H_2_O_2_ reduction. H_2_O_2_ is a reactive oxygen species that can cause oxidative stress resulting in cellular damage.^[^ ^30^, ^31^ ^]^ Therefore, it was necessary to investigate the possible accumulation of this deleterious intermediate at the modified electrode surface. For this, a horseradish peroxidase (HRP)‐based microbiosensor was used, ensuring the selective and sensitive detection of H_2_O_2_. The enzyme HRP was embedded in an Os‐complex‐modified redox polymer (P‐Os, Figure S3) and immobilized on a carbon‐based microelectrode. Cyclic voltammograms performed with the HRP‐based microbiosensor in the absence of H_2_O_2_ revealed the characteristic wave of P‐Os, with a midpoint potential of around 430 mV versus SHE (Figure S4). After the addition of 1.0 mM H_2_O_2_ to the electrochemical cell, a pronounced cathodic catalytic response was observed, verifying the enzymatic H_2_O_2_ conversion mediated by P‐Os. As depicted in Figure S5A, the HRP‐modified microelectrode was used as a probe in an SECM setup for the analysis of an electrode modified with cyanobacterial cells embedded in P‐vio (sample). The sample was monitored while applying two different potentials. Initially, a potential of 200 mV versus SHE was applied. Under such conditions, P‐vio remains oxidized and cannot consume O_2_. Subsequently, a potential of −500 mV versus SHE was applied to the sample for reducing P‐vio and activating O_2_ consumption. During the experiment, the HRP‐based microbiosensor was used for H_2_O_2_ detection, recording its reduction at an applied potential of 200 mV versus SHE. The obtained results revealed only a minimal response associated with H_2_O_2_ detection with the microbiosensor, accounting for only 0.2 nA, when the negative potential was applied to the sample for 250 s (Figure S5B). This observation confirmed that the consumption of O_2_ by P‐vio resulted in negligible H_2_O_2_ accumulation, and hence, the proposed O_2_‐scavenging film could be safely applied for photoH_2_ production with cyanobacterial cells.

### Successful O_2_ Depletion Enables PhotoH_2_ Production

**Figure 3:**
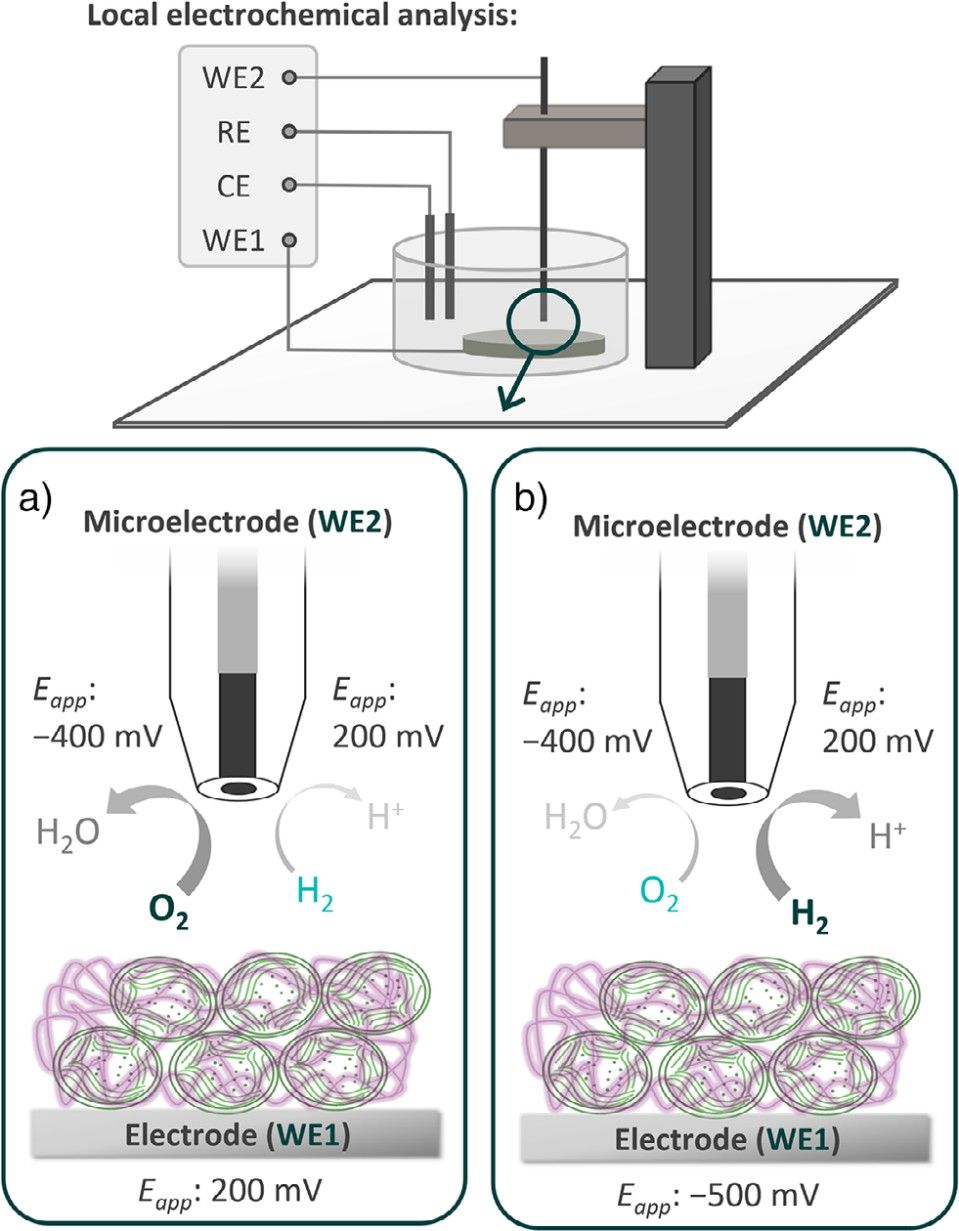
Schematic representation of the setup used for local electrochemical characterization of cyanobacterial cells embedded in P‐vio. A Pt microelectrode is used as a probe for the detection of O_2_ (reduction at −400 mV) or H_2_ (oxidation at 200 mV). a) When the sample is polarized at 200 mV the O_2_ protection system is switched off. b) O_2_ protection system switched on by polarizing the sample at −500 mV. All applied potentials (*E_app_*) versus SHE. Objects not drawn to scale. For simplicity, the light source is omitted from the setup and the stoichiometry of the species converted at the microelectrode is not balanced. CE: counter electrode, RE: reference electrode, WE1: working electrode 1 (glassy carbon electrode modified with cyanobacterial cells embedded in P‐vio), WE2: working electrode 2 (Pt microelectrode).

**Figure 4:**
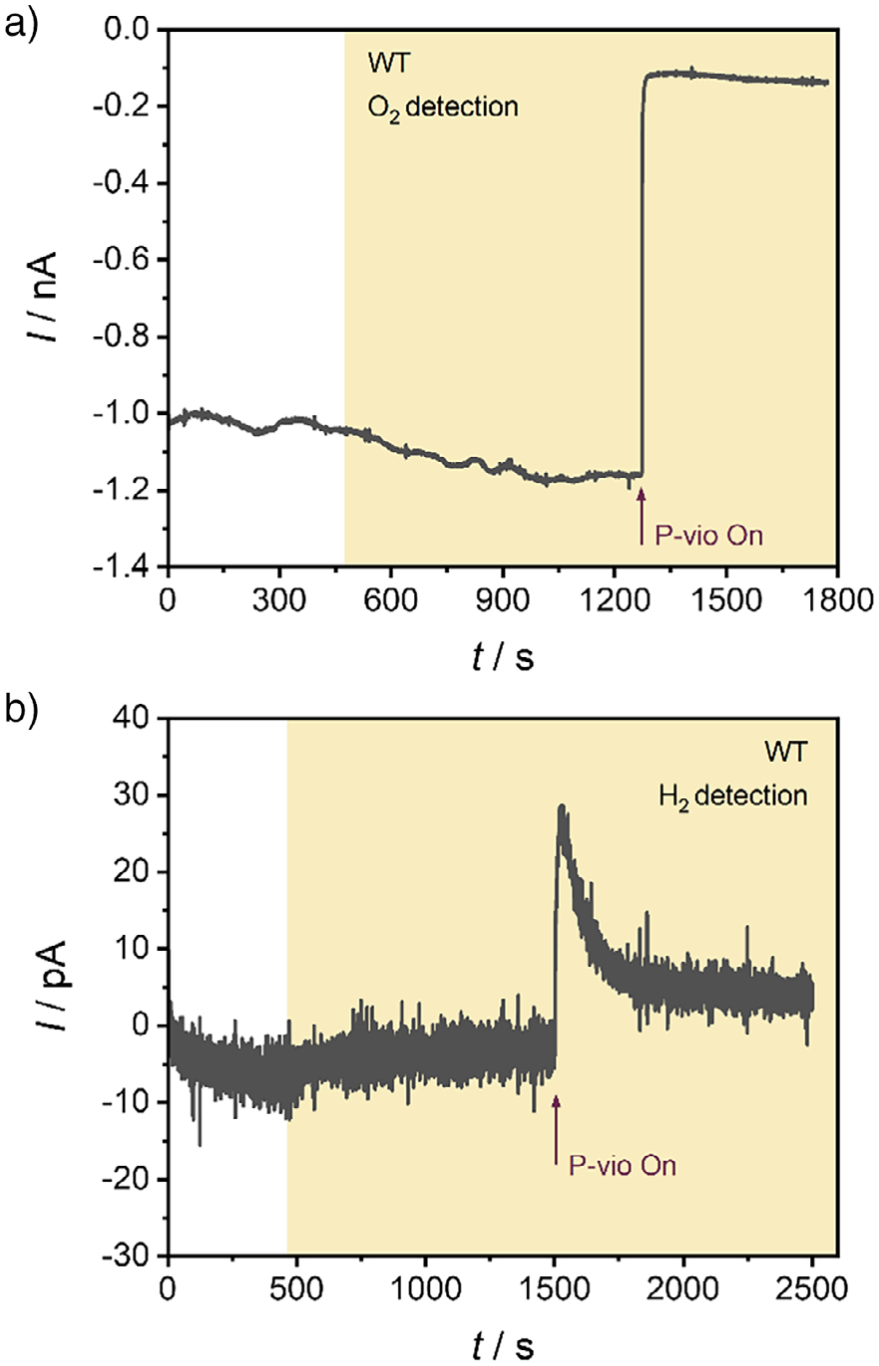
Local electrochemical analysis of a glassy carbon electrode modified with WT cyanobacterial cells embedded in P‐vio. The sample was initially polarized at 200 mV under dark, illuminated using white light (17 mW cm^−2^, as indicated by the yellow background), and subsequently polarized at −500 mV for P‐vio reduction (at the time indicated by the arrows), switching on the O_2_ removal system. a) Cathodic current for O_2_ reduction with the positioned Pt microelectrode at a potential of −400 mV. b) Anodic current for H_2_ oxidation with the positioned Pt microelectrode at a potential of 200 mV (all potentials versus SHE). Electrolyte: Ar‐saturated 0.1 M phosphate buffer, pH 7.0. Tip‐to‐sample distance: 20 µm.

The system was further investigated using SECM to monitor O_2_ release from the immobilized living cells performing photosynthesis under light conditions. A Pt microdisk electrode was used as an electrochemical probe, which enabled the straightforward detection of reaction products in a localized space (Figure 3). O_2_ was detected by its electrochemical reduction at the Pt microdisk electrode. WT cyanobacterial cells integrated within P‐vio were initially investigated at an applied potential of 200 mV versus SHE (Figure 3a). As depicted in Figure 4a, a background O_2_ reduction current was recorded at the Pt microelectrode, even after the electrolyte solution was purged with Ar to remove dissolved O_2_. This could be explained by O_2_ remaining in the polymer film during electrode preparation. After the sample was illuminated, an increase in the cathodic response at the Pt microelectrode was observed, indicating photosynthetic O_2_ evolution from the immobilized cells. Next, the O_2_‐removal system was switched on by applying a negative potential to the P‐vio film (Figure 3b), which translated to a sudden drop in the microelectrode cathodic current associated with O_2_ detection. Under such circumstances, P‐vio was reduced which in turn allows comsumption of molecular oxygen. This result suggested that incorporating cyanobacterial cells within the viologen‐functionalized redox polymer allows for efficient O_2_ removal, in close proximity to the location of the cyanobacterial cells.

The microelectrode was also used to assess H_2_ evolution from the investigated samples, following the H_2_ oxidation current at the Pt surface. As shown in Figure 4b, only a minimal response for H_2_ evolution was observed upon sample illumination. This was expected as a result of O_2_ produced by the cells under illumination, negatively impacting the activity of H_2_ase for H_2_ production (as schematically depicted in Figure 3a).^[^ ^12^ ^]^ After a suitable potential for P‐vio reduction was applied, switching on O_2_ removal, a fast rise in the microelectrode current for H_2_ detection was observed (see also Figure 3b). This correlated with the rapid O_2_ depletion measured before and confirmed the possibility for photoH_2_ production with WT cyanobacterial cells. Under this situation, H_2_ase can be rapidly reactivated by electrons received from the photosynthetic chain.^[^ ^15^ ^]^ Nonetheless, photoH_2_ production decreased exponentially over time, even though sustained O_2_ depletion was observed (see Figure 4a). The obtained results highlighted the possibility of attaining the required conditions for practical photoH_2_ production, using the proposed electrochemical strategy for O_2_ protection of cyanobacterial cells. However, it was also clear that WT cells have a limited H_2_ evolution performance, which is heavily hampered by H_2_ uptake by the native bidirectional HoxEFUYH hydrogenase.^[^ ^14^ ^]^

### Sustained PhotoH_2_ Production

**Figure 5:**
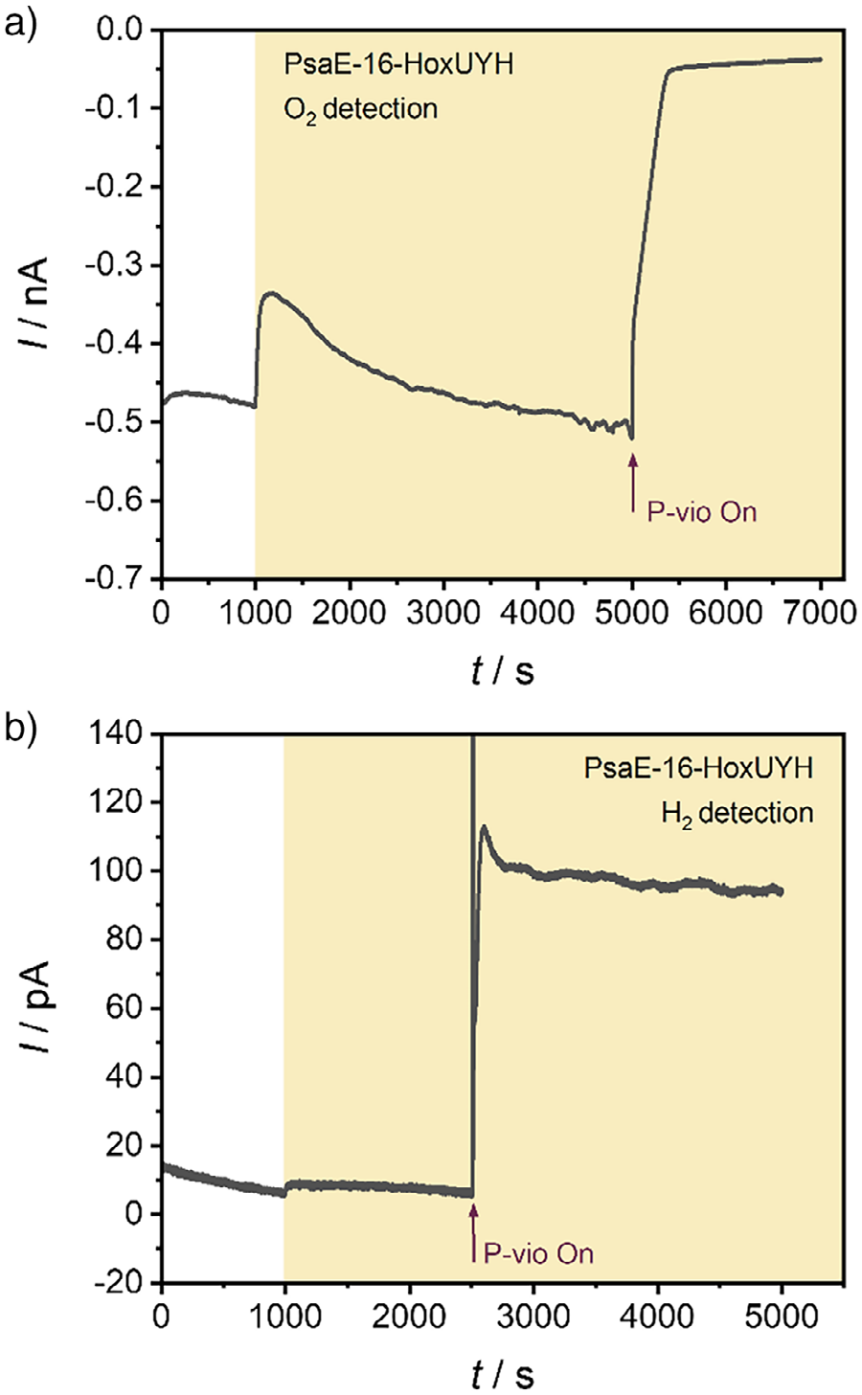
Local electrochemical analysis of a glassy carbon electrode modified with PsaE‐16‐HoxUYH cyanobacterial cells embedded in P‐vio. The investigated sample was initially polarized at 200 mV under dark, illuminated using white light (17 mW cm^−2^, as indicated by the yellow background), and subsequently polarized at −500 mV for P‐vio reduction (at the time indicated by the arrows), switching on the O_2_ removal system. a) Cathodic current for O_2_ reduction with a Pt microelectrode at a potential of −400 mV. b) Anodic current for H_2_ oxidation with the Pt microelectrode at a potential of 200 mV (all potentials versus SHE). Electrolyte: Ar‐saturated 0.1 M phosphate buffer, pH 7.0. Tip‐to‐sample distance: 20 µm.

Considering the aforementioned constraints of WT cyanobacterial cells for photoH_2_ production, the PSI–H_2_ase fusion mutant PsaE‐16‐HoxUYH was investigated in combination with the proposed electrochemical O_2_ protection system. PsaE‐16‐HoxUYH cells were incorporated into the P‐vio redox polymer and immobilized on a glassy carbon electrode, as done before with WT cells. Following the O_2_ reduction current at the Pt microelectrode, the PsaE‐16‐HoxUYH/P‐vio sample was examined under different conditions (Figure 5a). An initial period under dark showed a background reduction current at the microelectrode, indicating the presence of some O_2_ near the film even when the solution was purged with Ar to remove dissolved O_2_. This behavior was similar to that of WT cells and could be explained by O_2_ trapped in the film during electrode preparation. Upon illumination, a sharp decrease in the cathodic current at the microelectrode was observed. Subsequently, and as illumination proceeds for some time, the O_2_ concentration near the film was observed to increase, as a result of photosynthesis taking place leading to the release of O_2_ associated with water oxidation. When O_2_ scavenging was switched on by applying a negative potential for P‐vio reduction, the cathodic current at the Pt microelectrode declined significantly. This was linked to a constant reduction current of the P‐vio film (see Figure S6) and confirmed that O_2_ could be scavenged to a great extent, even when the immobilized cells were continuously performing photosynthesis.

The PsaE‐16‐HoxUYH/P‐vio sample was also investigated while detecting H_2_ (Figure 5b). As before, the sample was initially characterized at an applied potential where P‐vio remains oxidized and therefore cannot consume O_2_. When the sample was illuminated, a small increase in anodic current at the Pt microelectrode was observed, indicating residual H_2_ production with the PsaE‐16‐HoxUYH cells. When the O_2_ scavenging system was activated by applying a more negative potential to P‐vio, a fast increase in the H_2_ detection signal was recorded, which confirmed an increased rate for photoH_2_ production when O_2_ was depleted from the film. The obtained results are consistent with those for WT cells and also in agreement with observations made with PsaE‐16‐HoxUYH cells in liquid cultures. Interestingly, photoH_2_ production with PsaE‐16‐HoxUYH cells into P‐vio was observed to remain at high rates for much longer time periods in comparison with WT cells. For WT cells, the H_2_ detection signal decayed more than 75% after 1000 s of the maximum observed when switching on P‐vio (Figure 4b). In contrast, for PsaE‐16‐HoxUYH cells, the H_2_ signal decayed only 15% after 2500 s (Figure 5b), highlighting a more efficient H_2_ production in the mutant cells. This confirmed sustained photoH_2_ production with cyanobacterial cells using the proposed electrochemical protection system for scavenging molecular oxygen.

## Conclusions

The implementation of a functional strategy for photoH_2_ production in vivo using cyanobacterial cells was demonstrated. O_2_ produced during photosynthesis imposes an undesirable environment for photoH_2_ production within the cell, as it compromises H_2_ase activity for H_2_ evolution. In conventional approaches for photoH_2_ production over long time periods in liquid cultures, O_2_ is scavenged by the addition of glucose, glucose oxidase, and catalase. This procedure has the disadvantage that glucose is metabolized by the cells and becomes an undesired electron donor to photoH_2_ production, in addition to the desired electron donation solely by water splitting at PSII. Our aim was to establish a strategy for efficient O_2_ removal, without the need to add glucose, by embedding intact cyanobacterial cells into a viologen‐modified redox polymer. Reduced viologen generated at a suitable applied potential efficiently scavenges molecular oxygen, depleting O_2_ from the environment surrounding the cells (see Figure 1). This proceeds without a noticeable accumulation of deleterious H_2_O_2_. The analysis of O_2_ in the vicinity of immobilized cells using scanning electrochemical microscopy demonstrated that the O_2_ removal system not only scavenged O_2_ from the electrolyte, but also O_2_ produced while performing photosynthesis. Because O_2_ is continuously consumed within the film, it is hypothesized that a steep concentration gradient across the cell membrane is established, which imposes a fast O_2_ flux leaving the immobilized cells. As a result, the required anaerobic environment for catalytic activity of H_2_ase is ensured in the light in parallel to photosynthesis, leading to sustained photoH_2_ evolution, which was proven by the local detection of H_2_ generated under illumination. The use of the PSI–H_2_ase fusion mutant PsaE‐16‐HoxUYH revealed enhanced and prolonged photoH_2_ production performance due to a minimized H_2_ uptake and a stable connection between PSI and H_2_ase as compared to wild‐type cells. Based on the experimental setup and the absence of externally added glucose, it can be concluded that unlike in previous approaches, the electrons for the prolonged photoH_2_ production in the PSI–H_2_ase fusion mutant most likely originate from photosynthetic water splitting at PSII. Next steps will be directed to a detailed characterization and optimization of the system and the design of a large‐scale biophotovoltaic device for photoH_2_ production using cyanobacterial cells.

## Supporting Information

The authors have cited additional references within the Supporting Information.^[^ ^32^, ^33^, ^34^ ^]^

## Conflict of Interests

The authors declare no conflict of interest.

## Supporting information



Supporting Information

## Data Availability

The data that support the findings of this study are available from the corresponding author upon reasonable request.
