# Supplementary material for: Electrochemical Protection of Cyanobacterial Cells from Molecular Oxygen Enables Sustained PhotoH2 Production
Source: Angew Chem Int Ed Engl. 2026 Jan 2;65(7):e19077. doi: 10.1002/anie.202519077 (PMC12887603; doi:10.1002/anie.202519077)
Supplement: Supplementary file 1 — Supporting Information [file ANIE-65-e19077-s001.docx]

**– Supporting Information –**

Electrochemical Protection of Cyanobacterial Cells from Molecular Oxygen Enables Sustained PhotoH_2_ Production

Panpan Wang,^[a]^ Florian Paul,^[b]^ Marko Boehm,^[b]^ Jens Appel,^[b]^ Kirstin Gutekunst,*^[b]^ Wolfgang Schuhmann,*^[a]^ Felipe Conzuelo,*^[c]^

^[a]^ Analytical Chemistry – Center for Electrochemical Sciences (CES), Faculty of Chemistry and Biochemistry, Ruhr University Bochum, Universitätsstr. 150, D-44780 Bochum, Germany

^[b]^ Molecular Plant Physiology, Bioenergetics in Photoautotrophs, University Kassel, Heinrich-Plett-Straße 40, D-34132 Kassel, Germany

^[c]^ Instituto de Tecnologia Química e Biológica António Xavier, Universidade Nova de Lisboa, Av. da República, 2780-157 Oeiras, Portugal

*E-mail: kirstin.gutekunst@uni-kassel.de, wolfgang.schuhmann@rub.de, felipe.conzuelo@itqb.unl.pt

**Contents Page**

Experimental Section S2

Supporting Figures S4

References to the Supporting Information S6

**Experimental Section**

*Chemicals and Materials*

All chemicals were of laboratory grade or higher and used as received, without further purification. Potassium dihydrogen phosphate and di-potassium hydrogen phosphate trihydrate were obtained from VWR Chemicals. Branched polyethyleneimine (PEI, average molecular weight: 600 g mol^−1^) and peroxidase from horseradish (HRP, ≥ 250 U mg^−1^ solid) were obtained from Sigma-Aldrich. Hydrogen peroxide was obtained from Fisher Chemicals. All solutions were prepared using deionized water (ρ = 18 MΩ cm) from a water purification system (SG Water).

The synthesis and purification of the viologen-modified polymer, poly(3-azidopropyl methacrylate-*co*-butyl acrylate-*co*-glycidyl methacrylate)-viologen (P-vio), can be found in ref.^[1]^ A detailed description of the synthesis and purification of the redox polymer poly(1-vinylimidazole-*co*-allylamine)-[Os(2,2’-bipyridine)_2_Cl]Cl (P-Os, Figure S3) has been reported before.^[2]^

*Growth Conditions of Cyanobacteria*

Wild-type cells of *Synechocystis* sp. PCC 6803 or the mutant PsaE-16-HoxUYH were grown at 28 °C and 50 µE m^–2^ s^–1^ on BG-11 agar plates and in bubbling cultures. To prepare cells for the *in vivo* experiments, embedding cells into a redox polymer, the cultures were incubated up to an OD_750_ = 1.0, as 200 mL bubbling cultures for about 3 days, and then harvested at 3000 rcf (24 °C).

*Simultaneous Measurements of H_2_ and O_2_*

An isotope-ratio mass spectrometer (IR-MS) Delta Q from ThermoFisher was used to monitor O_2_, while H_2_ concentrations were measured with a H_2_ sensor from Unisense. The inlet system for the mass spectrometer was home-made and contained a 1×1 cm^2^ quartz-cuvette with a removed bottom, mounted on top of a PTFE membrane (standard membrane, Yellow Springs Instrument) that was connected to the MS. On top of the sample, a plastic stopper was inserted that accommodated the guide of the H_2_ sensor so that the microelectrode could be inserted into a small capillary opening to reach the sample. The volume inside the cuvette was constantly stirred by a magnetic bar. An argon trace was used to reduce the noise of the O_2_ trace and to correct for gas consumption by the MS. To this end, the concentration decline of O_2_ for samples without cells was measured repeatedly before and found to be a fixed ratio to the consumption rate of Ar. These ratios, in combination with the actual O_2_ concentration, were used to calculate the consumption rate for each data point. H_2_ consumption by the MS was also recorded in samples without cells and used to correct the H_2_ turnover rates. Cell density was adjusted to 10 µg chlorophyll mL^–1^. To induce anaerobiosis, glucose oxidase (40 U mL^–1^) and catalase (50 U mL^–1^) were added, together with 2 mM glucose.

*Electrode Modification*

Glassy carbon disk electrodes (*d* = 3 mm, CH Instruments) were cleaned by polishing with alumina slurries of different grain sizes following standard protocols. Afterwards, the electrodes were modified with 6.5 µL of a mixture of 1.0 µL P-vio (13.5 mg mL^−1^), 5.0 µL of a suspension of WT cells (OD_680_ = 1.0) or PsaE-16-HoxUYH mutant cells (OD_680_ = 2.0), and 0.5 µL PEI (1.0 mg mL^−1^). The modified electrodes were incubated at 30  C in the dark for 30 min before electrochemical characterizations were performed.

*Electrochemical Characterizations*

Local detection of light-induced reaction products was performed by scanning electrochemical micros­copy (SECM). SECM measurements were performed using the electrodes modified with cyanobacterial cells embedded in P-vio as a sample, a Ag/AgCl/3 M KCl reference electrode, and a Pt cylindrical mesh as the counter electrode. A Pt disk microelectrode (*d* = 25 µm) was used as an SECM probe. The SECM setup consisted of step-motor-driven micrometer screw (Owis) and a piezoelectric positioner (NanoCube, Physik Instrumente) for accurate positioning of the microelectrode in x–y–z directions, a bipotentiostat (PGU-BI 100, IPS-Jaissle), and an in-house written control software. Measurements were performed with the microelectrode positioned at a constant distance of 20 µm above the investigated sample surface, in a stationary configuration. For O_2_ detection, the Pt microelectrode was polarized at a potential of −400 mV vs. SHE. H_2_ detection was performed with the Pt microelectrode at a potential of 200 mV vs. SHE. A He-Xe lamp (LC8 type 03, Hamamatsu Photonics) was used for illumination with white light at a measured incident power of 17 mW cm^−2^. All measurements were performed at room temperature.

For H_2_O_2_ detection, a carbon paste microelectrode was modified by drop-casting 5 × 1.0 µL of a mixture of HRP (5.0 mg mL^−1^) and P-Os (2.5 mg mL^−1^).^[3]^ The modified microelectrode was incubated at 4 °C overnight before use.

**Supporting Figures**


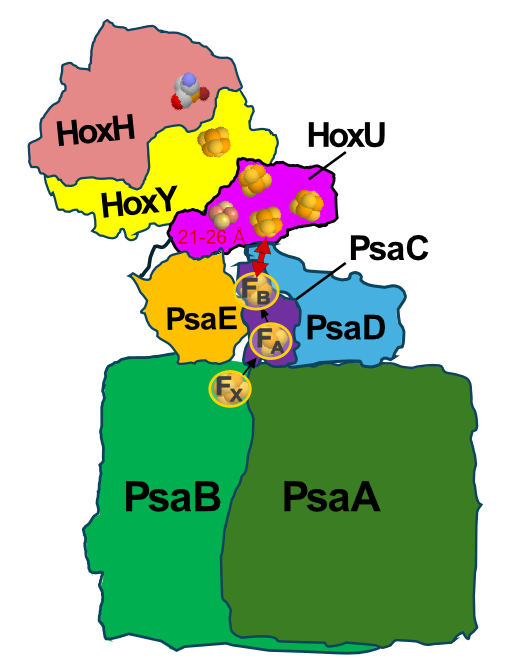


**Figure S1.** Schematic representation of the PSI-H_2_ase fusion expressed in the PsaE-16-HoxUYH mutant.


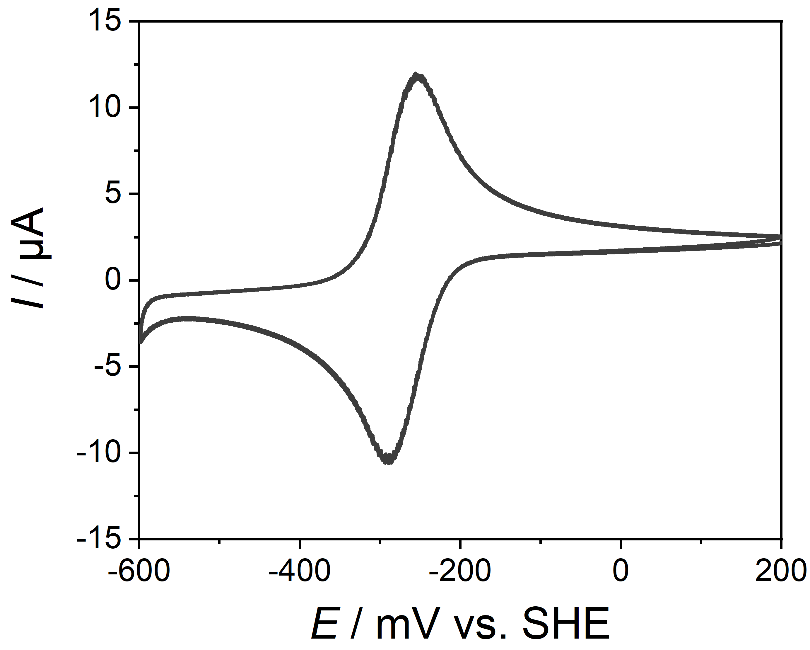


**Figure S2.** Voltammetric response for a glassy carbon electrode modified with WT cyanobacterial cells into a P-vio film. Electrolyte: Ar-saturated 0.1 M phosphate buffer, pH 7.0. Dark conditions. Three consecutive cycles. Scan rate: 10 mV s^−1^.

**Figure S3.** Chemical structure of the redox polymer poly(1-vinylimidazole-*co*-allylamine)-[Os(2,2’-bipyridine)_2_Cl]Cl (P-Os).


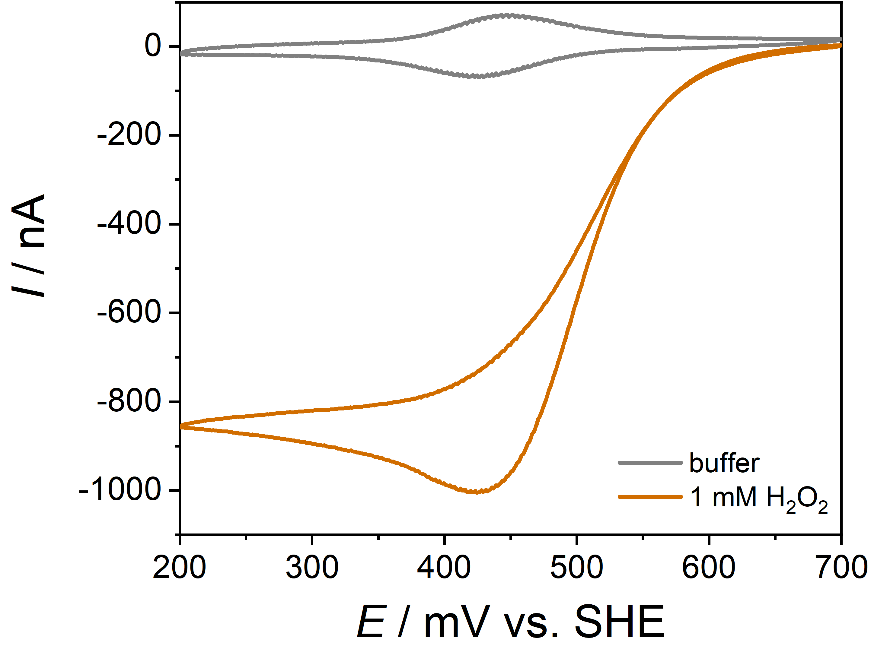


**Figure S4.** Voltammetric characterization of the HRP-modified microelectrode in the absence (gray) and presence (orange) of 1.0 mM H_2_O_2_. Electrolyte: 0.1 M phosphate buffer, pH 7.0. Scan rate: 10 mV s^−1^.


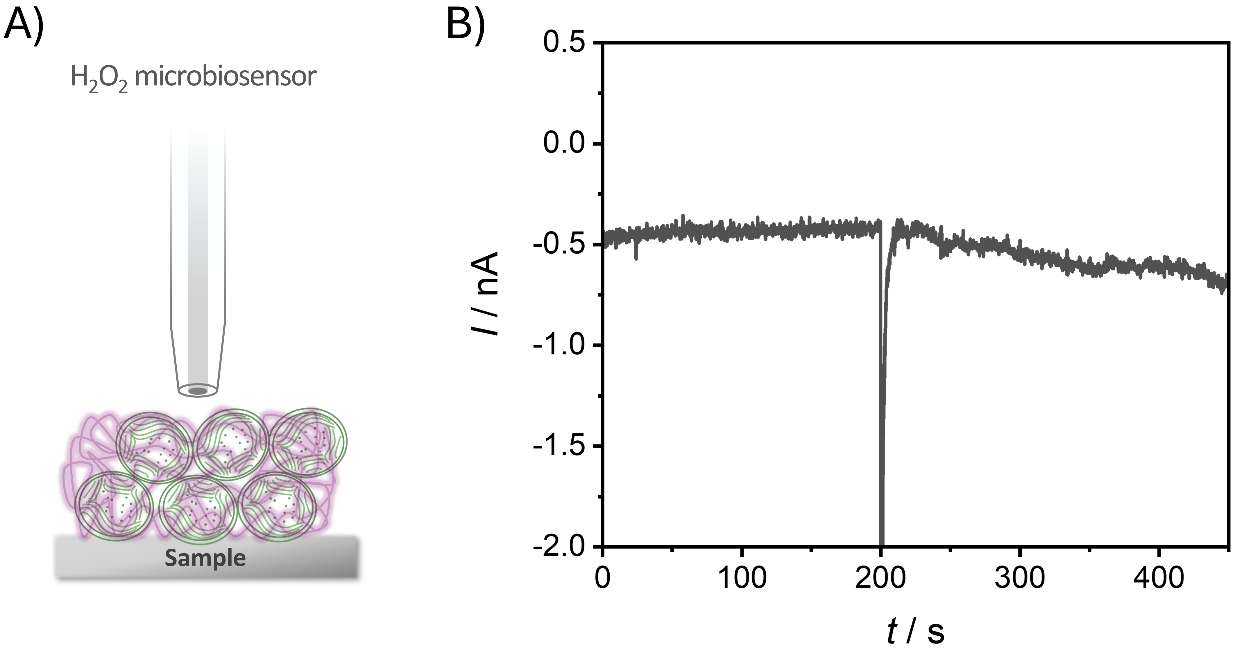


**Figure S5.** A) Schematic representation of H_2_O_2_ detection at a glassy carbon electrode modified with cyanobacterial cells (PsaE-16-HoxUYH) embedded in a P-vio film using an HRP-based microbiosensor as probe. B) Response recorded with the HRP microsensor. The sample was initially polarized at 200 mV vs. SHE. At *t* = 200 s, the applied potential was switched to −500 mV for the reduction of P-vio enabling O_2_ consumption. The microbiosensor was constantly polarized at 200 mV vs. SHE. Electrolyte: Ar-saturated 0.1 M phosphate buffer, pH 7.0. During the experiment, the sample was illuminated with white light (17 mW cm^−2^).


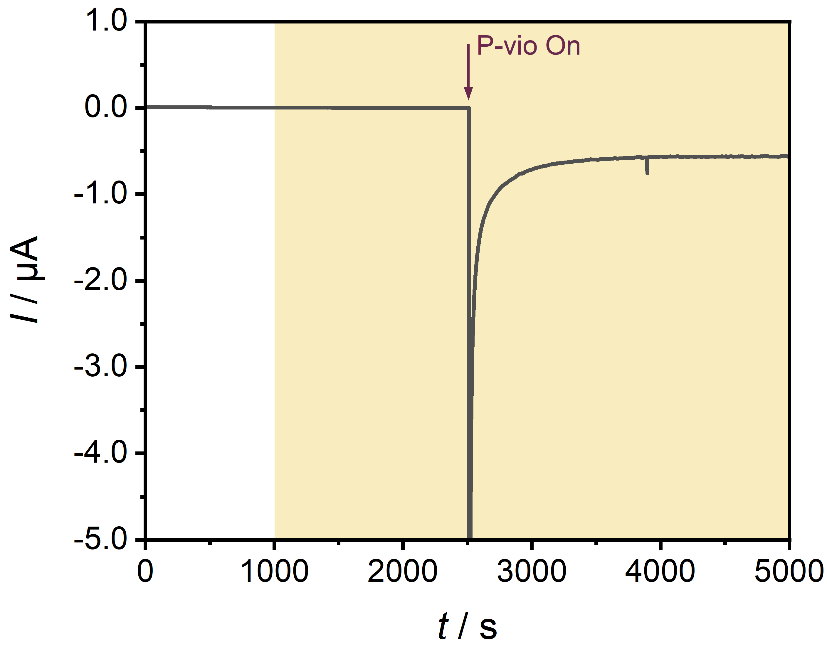


**Figure S6.** Current recorded with a glassy carbon electrode modified with PsaE-16-HoxUYH cells embedded into P-vio. The electrode was initially polarized at 200 mV under dark, illuminated using white light (17 mW cm^−2^, as indicated by the yellow background), and subsequently polarized at −500 mV (at the time indicated by the arrow) for P-vio reduction and O_2_ removal. All potentials vs. SHE. Electrolyte: Ar-saturated 0.1 M phosphate buffer, pH 7.0.

**References to the Supporting Information**

[1] F. Zhao, P. Wang, A. Ruff, V. Hartmann, S. Zacarias, I. A. C. Pereira, M. M. Nowaczyk, M. Rögner, F. Conzuelo, W. Schuhmann, "A photosystem I monolayer with anisotropic electron flow enables Z-scheme like photosynthetic water splitting" *Energy Environ. Sci.* **2019**, *12*, 3133.

[2] F. Conzuelo, N. Marković, A. Ruff, W. Schuhmann, "The open circuit voltage in biofuel cells: Nernstian shift in pseudocapacitive electrodes" *Angew. Chem. Int. Ed.* **2018**, *57*, 13681.

[3] F. Zhao, F. Conzuelo, V. Hartmann, H. Li, S. Stapf, M. M. Nowaczyk, M. Rögner, N. Plumeré, W. Lubitz, W. Schuhmann, "A novel versatile microbiosensor for local hydrogen detection by means of scanning photoelectrochemical microscopy" *Biosens. Bioelectron.* **2017**, *94*, 433.
